# Prospective randomized pharmacogenetic study of topiramate for treating alcohol use disorder

**DOI:** 10.1038/s41386-020-00945-9

**Published:** 2021-02-10

**Authors:** Henry R. Kranzler, Paige E. Morris, Timothy Pond, Richard C. Crist, Kyle M. Kampman, Emily E. Hartwell, Kevin G. Lynch

**Affiliations:** 1 Department of Psychiatry, University of Pennsylvania Perelman School of Medicine, Philadelphia, PA 19104 USA; 2 Mental Illness Research, Education and Clinical Center, Veterans Integrated Service Network 4, Crescenz VAMC, Philadelphia, PA 19104 USA

**Keywords:** Medical research, Diseases

## Abstract

In a prior study, topiramate reduced heavy drinking among individuals who sought to reduce their drinking, with the effect moderated by a single nucleotide polymorphism (SNP; rs2832407) in *GRIK1*, which encodes the kainate GluK1 receptor subunit (Kranzler et al. 2014). The present study sought to replicate prospectively the effect of topiramate and rs2832407 in patients with DSM-5 alcohol use disorder (AUD) who sought to reduce or stop their drinking. We stratified the randomization on genotype (rs2832407*C-allele homozygotes vs. A-allele carriers) and assigned 170 European-American participants (71.2% male) to receive 12 weeks of treatment with topiramate (*N*  = 85), at a maximal daily dosage of 200 mg, or matching placebo (*N* = 85). At each of nine treatment visits participants received brief counseling to reduce drinking and increase abstinent days. We hypothesized that topiramate-treated patients with the rs2832407*CC genotype would reduce heavy drinking days (HDDs) more than the other three groups. The rate of treatment completion was 91.8% in both groups. The mean number of HDDs per week in the placebo group was 1.67 (95% CI = (1.29, 2.16), *p* = 0.0001) times greater than in the topiramate group, which was confirmed by the topiramate group’s significantly greater reduction in the concentration of the liver enzyme γ-glutamyltransferase and lower alcohol-related problems score. There was no significant difference in topiramate’s effect on HDDs between genotype groups. Although consistent with other studies showing a reduction in heavy drinking with topiramate treatment, the prior finding of a moderating effect of rs2832407 genotype was not replicated in this prospective trial.

## Introduction

Heavy drinking is common in the United States: in 2018, 6.6% of adults reported heavy alcohol use (consuming five or more drinks for males or four or more drinks for females on five or more days during the preceding month) [1]. From 2006 to 2010, there were ~88,000 annual alcohol-associated deaths in the US, 9.8% of all US deaths [2]. Despite these risks, only a small fraction of heavy drinkers in the population receive any kind of alcohol treatment, with medications particularly underutilized [3].

One of the medications that has been studied for treating alcohol use disorder (AUD) is topiramate. In the most comprehensive meta-analysis of effects of topiramate on alcohol consumption to date [4], the drug was associated with clinically significant effects (Hedges *g* > 0.4) on promoting abstinence and reducing heavy drinking and smaller effects (Hedges *g* > 0.3) on reducing γ-glutamyltransferase (GGT) concentration and alcohol craving.

Topiramate is an antagonist of kainate receptors [5, 6], where it is most potent and selective for receptors containing the GluK1 subunit (encoded by *GRIK1)* [7, 8]. We examined the association of single nucleotide polymorphisms (SNPs) in *GRIK1* to the risk of alcohol dependence [9]. Of the seven SNPs examined only rs2832407, a C-to-A non-coding substitution, was significantly associated with alcohol dependence. In a subsequent randomized, controlled trial (RCT), we found that individuals homozygous for the rs2832407*C allele reduced their heavy drinking significantly more than A-allele carriers treated with topiramate or placebo-treated subjects [10], though in that study the randomization was not stratified by genotype.

To validate both the pharmacogenetic findings and the efficacy of the 200 mg/d dosage, we conducted an RCT in a larger sample using a prospective pharmacogenetic design, in which C-allele homozygotes and A-allele carriers were randomized separately to receive topiramate or placebo for 12 weeks of treatment. We hypothesized that topiramate-treated patients would show a greater reduction in heavy drinking days (HDDs) than the placebo group and that rs2832407 would moderate this effect.

## Patients and methods

### Overview

The study was a parallel-groups, placebo-controlled trial of topiramate in heavy drinkers, all of whom received medical management [11], a brief psychosocial intervention weekly for the first six weeks and then biweekly for six weeks. Patients were randomly assigned equally to treatment groups by staff who were not involved in providing treatment, using a random allocation stratified on rs2832407 genotype provided by the statistician. Double-blind treatment conditions were maintained throughout the study. Raters were trained in the reliable use of all assessments. The study included a 2-week pre-treatment assessment period, a 12-week treatment period, and a 1-week medication taper period.

### Patients

Inclusion criteria were age 18–70; self-identified European ancestry; a weekly average of ≥24 standard drinks (men) or ≥18 standard drinks (women), which exceed empirically-based moderate drinking limits [12]; a current DSM-5 diagnosis of AUD; a goal of either stopping drinking or reducing their drinking; ability to read English minimally at an 8^th^ grade level; no gross evidence of cognitive impairment; and willingness to provide written, informed consent to participate and to name someone who could be contacted to locate the patient if he or she could not be reached. In addition, women of childbearing potential had to be nonlactating, practicing a reliable method of birth control, and provide a negative urine pregnancy test at the screening and randomization visits.

Prospective patients were excluded based on the presence of a current, clinically significant physical disease or abnormality on medical history, physical examination, or routine laboratory evaluation; a history of nephrolithiasis, glaucoma, or hypersensitivity to topiramate; treatment with carbonic anhydrase inhibitors or dolutegravir; a serious psychiatric illness (i.e., schizophrenia, bipolar disorder, severe or psychotic major depression, panic disorder, borderline or antisocial personality disorder, organic mood or mental disorders, eating disorder, or imminent suicide or violence risk) or current treatment with a psychotropic medication or one aimed at reducing drinking; a current DSM-IV diagnosis of non-nicotine drug dependence; a urine drug screen positive for opioids, cocaine, benzodiazepine, barbiturates, or amphetamines; or a clinical presentation that was judged by a physician to require abstinence from alcohol for safety reasons (e.g., current gastritis, a recent or past history of severe alcohol withdrawal symptoms).

### Procedures

The study was conducted from December 29, 2014 through August 1, 2019 at the University of Pennsylvania Treatment Research Center (Penn; *n* = 164) and the Corporal Michael J. Crescenz Veterans Affairs Medical Center (CMCVAMC; *n* = 6), which corresponded to the duration of funding. The institutional review board at each site approved the study protocol. Patients gave written informed consent to participate and were paid to complete research assessments and return study medication bottles for capsule counts.

At Penn, subjects were recruited through media advertisements and at the CMCVAMC through clinician referrals and the screening of medical records. All potentially eligible participants underwent an initial 20-min telephone screening interview. Eligible candidates were seen for an in-person visit, where they gave written, informed consent to participate and underwent a medical history, physical examination, clinical laboratory testing, a urine drug screen, and pregnancy testing and gave a blood sample for DNA extraction and genotyping.

Using data from our prior study [10], we estimated that a sample size of 188 would provide power >0.80 for a two-sided test at *α* = 0.05. We screened 320 prospective participants in person and randomly assigned 170 (121 men, 71.2%) to treatment with topiramate or placebo. A CONSORT diagram (Supplementary Fig. S1) shows the reasons that 150 potential participants were excluded.

Prior to randomization, patients completed questionnaires and research interviews administered by a trained research evaluator. A study physician reviewed the study design and potential adverse events with all patients and answered any questions that they had. A nurse then delivered the first medical management session [11] and dispensed study medication. A separate block randomization scheme at each of the two sites balanced the medication groups within strata defined by genotype group (CC vs. AA/AC) and treatment goal (Reduce vs. Abstain), and these variables were included as factors in all analyses.

Following treatment initiation, patients were seen weekly for six weeks to gradually increase the dosage of medication, followed by three biweekly treatment visits. At each visit, we measured the patient’s breath alcohol concentration, weight, and vital signs; patients completed questionnaires; and the nurse elicited information on concurrent medications, emergent adverse events, and protocol adherence, and delivered the medical management intervention [11]. Patients were also interviewed to assess their drinking and medication usage since the last visit and the nurse compared self-reported adherence to the number of capsules returned, seeking to resolve any discrepancies with the patient. At the end of the 12-week treatment period, patients again completed questionnaires and were interviewed by the research nurse, the research evaluator, and the study physician.

### Study treatments

Counseling used a medical management manual that focuses on medication adherence and treatment participation consistent with the patient’s goal of abstinence or non-hazardous drinking. The initial session, which lasted 45 min, included a review of the results of the initial evaluation, A Guide to Sensible Drinking [13], and the rationale for pharmacotherapy. Subsequent sessions lasted 20–30 min, at which the nurse assessed the patient’s drinking and medication adherence through capsule counts and made recommendations on both. Based on guidelines for non-hazardous drinking [12], men were advised to consume no more than three standard drinks per day and 12 standard drinks per week and women no more than two drinks per day and eight drinks per week. Patients whose goal was abstinence were counseled to avoid drinking completely. We audiotaped sessions and reviewed a sample of them and provided feedback to the three nurses to ensure consistency in their delivery of the counseling.

#### Medication

We chose a target dosage of 200 mg/day of topiramate as in our previous trial [10]. Placebo and topiramate were encapsulated and indistinguishable from one another. Medication was initiated at a dosage of 25 mg of topiramate (or one placebo capsule) daily for 1 week with weekly increases to a maximum of 100 mg twice daily during week six. We delayed a dosage increase or, if necessary, reduced the dosage to ameliorate adverse effects. At the end of treatment, the medication was gradually reduced to zero over a 1-week period.

### Assessments

Laboratory Testing included urinalysis, urine toxicology testing, urine pregnancy testing (as appropriate), a complete blood count, and a chemistry panel [comprised of electrolytes, liver transaminases, GGT, and bilirubin]. We repeated the serum bicarbonate concentration at the study midpoint to screen for metabolic acidosis and GGT at the study midpoint and endpoint to validate self-reported drinking.

Sociodemographic/Clinical Information was obtained at screening and included self-identified ancestry, marital status, educational and occupational information, medical history, and substance abuse treatment history.

Psychiatric Diagnoses were made using a modified version of the Structured Clinical Interview for DSM-IV (SCID-IV; [14]) that incorporated DSM-5 criteria [15] only for AUD. The SCID-IV was used to diagnose standard DSM-IV psychiatric disorders [16].

Alcohol Consumption was measured using the Timeline Follow-back Method [17] and included the number of drinking days (DDs) and HDDs during the 90-day pretreatment period and at each visit.

Alcohol-related Problems were measured using the Short Index of Problems [18], a 15-item measure that was administered at baseline and at the end of treatment using a three-month timeframe.

Depressive Symptoms were measured using the Patient Health Questionnaire [19], a 9-item self-report measure (score = 0–27) that was administered at baseline and at each study visit.

### Statistical analysis

#### Baseline comparisons

Descriptive statistics include means and standard deviations for continuous variables (group differences analyzed using *t*-tests) and counts for categorical variables (group differences analyzed using chi-square). Factorial models crossing treatment assignment with a two-level rs2832407 genotype group (CC vs. CA/AA) were analyzed using a linear model for continuous variables and logistic regression for binary variables.

### Timeline follow-back data

We examined two weekly drinking outcome measures: a primary measure—the number of days of heavy drinking (i.e., ≥4 drinks in a day for women and ≥5 drinks in a day for men)—and a secondary measure—the number of DDs.

Intent-to-treat analyses included all 170 patients. Number of weeks of treatment missed and of nonadherent weeks were compared using negative binomial models with robust standard errors. The analyses used generalized linear mixed models with a poisson distribution and log link function to examine medication group differences in changes in DDs and HDDs during treatment. The models included fixed effects for pre-treatment percent days heavy drinking (for the HDDs response) or pre-treatment percent days drinking (for the DDs response), treatment goal, genotype, medication group and week, and interactions between genotype (rs2832407*CC vs. CA/AA), medication group (topiramate vs. placebo), and week. Interactions involving group, genotype, and time were included to accommodate possible different group by time effects across genotypes. We repeated these analyses for the DD and HDD responses with genotype as a three-level variable (CC vs. AC vs. AA), as we had done in our prior study [10].

To examine the impact of missing data, we used pattern mixture models, with a binary variable indicating whether a participant completed the 12 weeks of treatment, or not, as a summary of missing data.

### Measures to validate drinking outcomes

Due to severe positive skewness and kurtosis the GGT values were log transformed and analyzed using linear mixed effects models. The SIP score was analyzed using ANCOVA, controlling for the pretreatment score.

### Adverse events

We used the Medical Dictionary for Regulatory Activities (https://www.meddra.org/) to categorize all adverse events (AEs).

## Results

### Demographic and pretreatment clinical measures (Table 1)

The study sample consisted predominantly of middle-aged (mean = 51.2 years), married (57.6%), employed (81.2%), men (71.2%), with a college education (mean = 16.2 years of education). During pretreatment, patients drank alcohol on a mean of 6.1 days per week, drinking heavily on 5.0 days per week. The lifetime prevalence of major depression was 21.5% and of an anxiety disorder 14.7%. There were no significant differences between the topiramate and placebo groups on any pretreatment demographic or clinical measures.

**Table 1:** Demographics and pretreatment clinical measures (*N* = 170).

Demographics	Topiramate (*N* = 85) *N* (% or SD)	Placebo (*N* = 85) *N* (% or SD)	*P* value
Sex (Male)	61 (72%)	60 (71%)	0.63
Age (yr)	52.3 (10.5)	50.0 (12.8)	0.20
Marital status^a^	47 (55%)	51 (60%)	0.27
Employment status			0.19
Full-time	51 (60%)	58 (68%)	
Part-time	13 (15%)	16 (19%)	
Not working	21 (25%)	11 (13%)	
Years of education	16.1 (2.4)	16.3 (2.5)	0.62
Annual income level			0.08
<$40,000	12 (14%)	11 (13%)	
$40,000–$79,999	15 (18%)	14 (16%)	
$80,000–$119,000	23 (27%)	29 (34%)	
≥$120,000	35 (41%)	31 (36%)	
Clinical measures			
Lifetime major depression	18 (21%)	19 (22%)	0.48
Lifetime anxiety disorder	9 (11%)	16 (19%)	0.08
PHQ-9 score	5.0 (3.9)	5.7 (4.3)	0.32
Drinking days^b^	87.2 (16.5)	86.3 (16.8)	0.72
Heavy drinking days^b^	73.8 (23.5)	68.7 (25.6)	0.18
SIP score^b^	14.2 (8.6)	14.3 (9.7)	0.98

*SD* standard deviation, *PHQ-9* patient health questionnaire-9, *SIP* short index of problems.

^a^Married or cohabiting.

^b^During the 90 days preceding the screening visit.

### Treatment completion

All 170 patients provided at least 1 week of timeline follow-back data during the 12-week treatment period. Overall, 158 of the 170 patients (92.9%) provided timeline data for all 12 study weeks; this included 81 (95.3%) of the placebo group and 77 (90.6%) of the topiramate group (*χ*^2^_(1)_ = 1.43, *p* = 0.23, OR = 2.10, 95% CI = (0.61, 7.27). In each medication group, 78 patients (91.8%) completed their week-12 visit. The medication groups were comparable on the number of weeks of treatment received [topiramate: mean = 10.6, SD = 3.0], placebo: mean = 10.9, SD = 3.0; *χ*^2^_(1)_ = 0.49, *p* = 0.49. The interaction of genotype group with medication group [*χ*^2^_(1)_ = 0.19, *p* = 0.67] and the main effect of genotype group [*χ*^2^_(1)_ = 0.45, *p* = 0.50] on weeks of treatment received were not significant.

### Medication adherence and maximal dosage achieved

Adherence rates were high in both medication groups: the placebo group averaged 6.6 d (SD = 1.5) of medication ingestion per week, while the topiramate group averaged 6.3 (SD = 2.0). The medication groups were comparable on the number of weeks of full adherence to treatment, defined as having taken medication on all seven days in a week [topiramate: mean = 9.3, SD = 3.3], placebo: mean = 10.4, SD = 3.1; *χ*^2^_(1)_ = 0.79, *p* = 0.37. The interaction of genotype group with medication group [*χ*^2^_(1)_ = 0.10, *p* = 0.75] and the main effect of genotype group [*χ*^2^_(1)_ = 0.43, *p* = 0.51] on weeks of treatment received were not significant. Fewer topiramate patients [59 (69.4%)] than placebo patients [78 (91.6%)] reached the maximal dosage (200 mg/day) or the equivalent number of placebo capsules [*χ*^2^_(1)_ = 13.57, *p* = 0.0002, OR = 4.91, 95% CI = (2.00,12.08)].

### Main effects of topiramate

#### Heavy drinking days (Fig. 1)

There were significant medication group by time effects (*F*_(1,1797)_ = 26.22, *p* < 0.001 for the linear term and *F*_(1,1797)_ = 6.26, *p* = 0.01 for the quadratic term), with topiramate patients reducing heavy drinking more than placebo patients. During treatment, the placebo group reported an average of 1.67 times more HDDs than the topiramate group (95% CI = (1.29, 2.16), *t*_1797_ = 3.87, *p* = 0.0001). During the last week of treatment, the number of HDDs in the placebo group was 1.80 (95% CI = (1.34, 2.41), *t*_1797_ = 3.95, *p* < 0.0001) times greater than in the topiramate group.

**Fig. 1:**
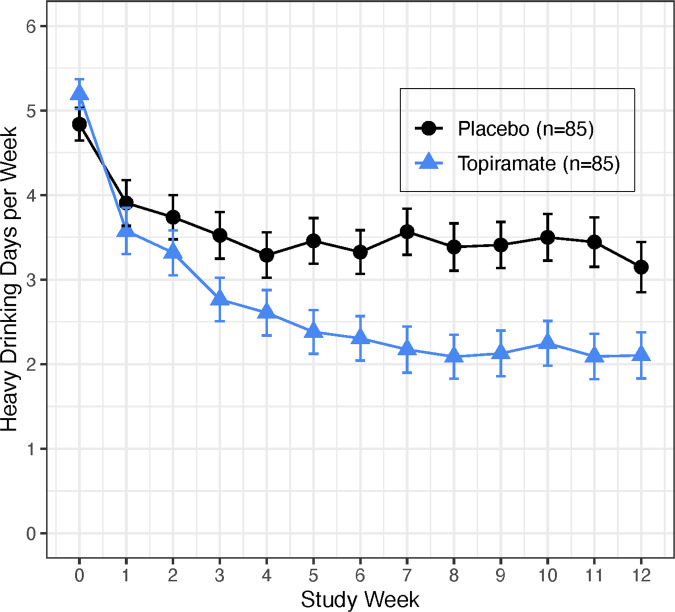
Mean (SEM) heavy drinking days per week by medication group. Significant medication group by time effects (*F*_(1,1797)_ = 26.22, *p* < 0.001 for the linear term and *F*_(1,1797)_ = 6.26, *p* = 0.01 for the quadratic term). The placebo group had an average of 1.67 times (95% CI = (1.29, 2.16), *t*_1797_ = 3.86, *p* = 0.0001) more heavy drinking days per week than the topiramate group.

#### Drinking days (Supplementary Fig. 2)

The medication group by time and quadratic time effects were non-significant. There was a significant (linear) effect of week (*F*_(1,1798)_ = 11.50, *p* = 0.0007), with the number of DDs decreasing over time in both groups. There was also a significant effect of medication group (*F*_(1,1798)_ = 4.52, *p* = 0.03), with placebo patients reporting an average of 1.16 [95% CI = (1.01, 1.33)] times more DDs per week than topiramate patients.

#### GGT concentrations

The medians and interquartile ranges for the distributions of GGT concentration in the placebo patients decreased from 40.0 IU/L [IQR = (22, 74)] at baseline to 31.0 IU/L [IQR = (19, 51)] at week 6 and 32.5 IU/L [IQR = (19, 55)] at endpoint, while the topiramate group decreased from from 37.5 IU/L (IQR = (24, 74)) at baseline to 31.0 IU/L [IQR = (19, 47)] at week 6 and 29.5 IU/L [IQR = (17, 44)] at endpoint. We compared the groups on the log10 scale, to account for the high levels of skewness in the raw GGT concentration distributions. Controlling for baseline concentrations, although there was not a significant treatment by time effect across visits week 6 and endpoint (*F*_(1,138)_ = 2.06, *p* = 0.14), there was a significant medication effect (*F*_(1,139)_ = 4.16, *p* = 0.04), with the log10 level among placebo patients being 0.044 [95% CI = (0.002, 0.086)] higher than those in the topiramate group.

#### Short index of problems (SIP) score

SIP scores decreased in the placebo group from 14.3 (SD = 9.9) to 9.7 (SD = 8.4) and in the topiramate group from 14.3 (SD = 8.6) to 7.2 (SD = 7.9). Controlling for baseline score, there was a significant medication effect on the SIP score at endpoint (*F*_(1,151)_ = 4.44, *p* = 0.04), with placebo patients’ scores being 2.2 points [95% CI = (0.16, 4.27)] higher than topiramate patients.

### Moderating effect of rs2832407 on the response to topiramate

Table 2 shows the demographic and pretreatment clinical features for the genotype by treatment groups. There were no demographic or clinical features that differed significantly among the groups.

**Table 2:** Baseline features by genotype and treatment assignment (*n* = 170).

Genotype group	CC (*N* = 61; 35.9%)		AC/AA (*N* = 109; 64.1%)		*P* values		
Medication group	Topiramate (*N* = 30)	Placebo (*N* = 31)	Topiramate (*N* = 55)	Placebo (*N* = 54)	Medication group	Genotype group	Interaction effect
Demographics							
Sex (Male)	22 (73%)	24 (77%)	39 (71%)	36 (67%)	0.86	0.35	0.56
Age (yr)^a^	53.0 (11.9)	47.9 (14.6)	51.9 (9.8)	51.2 (11.6)	0.20	0.57	0.27
Marital status^b^	16 (53%)	15 (48%)	31 (56%)	36 (67%)	0.52	0.18	0.33
Employment status					0.15	0.99	0.40
Full-time	20 (67%)	20 (65%)	31 (56%)	38 (70%)			
Part-time	2 (7%)	6 (19%)	11 (20%)	10 (19%)			
Not Working	8 (27%)	5 (16%)	13 (24%)	6 (11%)			
Education (yr)^a^	16.4 (2.3)	15.6 (2.9)	16.0 (2.4)	16.7 (2.1)	0.61	0.35	0.07
Annual income level					0.27	0.80	0.59
<$40,000	1 (3%)	8 (26%)	11 (20%)	3 (6%)			
$40,000–$79,999	8 (27%)	4 (13%)	7 (13%)	10 (19%)			
$80,000–$119,000	9 (30%)	8 (26%)	14 (25%)	21 (39%)			
≥$120,000	12 (40%)	11 (35%)	23 (42%)	20 (37%)			
Clinical measures							
Lifetime major depression	7 (23%)	11 (35%)	11 (20%)	8 (15%)	0.87	0.08	0.21
Lifetime anxiety disorder	5 (17%)	6 (19%)	4(7%)	10 (19%)	0.13	0.39	0.34
PHQ-9 score^a^	5.1 (3.4)	6.1 (4.1)	5.0 (4.2)	5.4 (4.5)	0.32	0.49	0.62
Drinking days^a,c^	86.6% (15.2)	85.6% (17.0)	87.5% (17.3)	86.9% (16.9)	0.71	0.71	0.98
Heavy drinking Days^a,c^	70.6% (22.8)	65.5% (26.2)	75.6% (23.9)	68.7% (25.6)	0.18	0.20	1.0
SIP score^a,c^	14.0 (7.9)	15.2 (10.3)	14.4 (9.1)	13.8 (9.4)	0.98	0.71	0.53

*PHQ-9* patient health questionnaire-9; *SIP* short index of problems.

^a^Values are mean and standard deviation.

^b^Married or cohabiting.

^c^During the 90 days preceding the screening visit.

#### Heavy drinking days

**Fig. 2:**
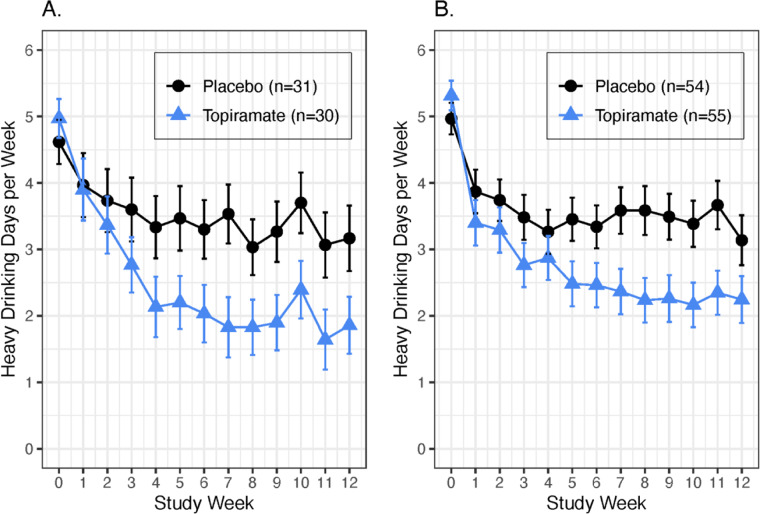
Mean (SEM) heavy drinking days per week by medication and genotype groups. **A** At endpoint, within the CC genotype patients, placebo patients had 1.74 times [95% CI = (1.13, 2.68)] more heavy drinking days per week than topiramate patients. **B** In the AC/AA genotype group, placebo patients had 1.63 times [95% CI = (1.18, 2.26)] more heavy drinking days per week than topiramate patients. The interaction effect was non-significant (*F*_(1,1797)_ = 0.05, *p* = 0.82).

At endpoint, within the CC genotype group, those receiving placebo reported 1.76 [95% CI = (1.22, 2.54)] times more HDDs per week than those treated with topiramate, compared to 1.63 [95% CI = (1.18, 2.33)] times more HDDs per week for placebo than topiramate within the AC/AA genotype group, a nonsignificant difference (*F*_(1,1797)_ = 0.05, *p* = 0.83) Fig. 2.

#### Drinking days (Supplementary Fig. 3)

Among CC genotype patients, those treated with placebo reported 1.29 [95% CI = (1.03, 1.62)] times more DDs per week than those receiving topiramate, compared to 1.09 [95% CI = (0.92, 1.29)] times more DDs per week in the AC/AA genotype group, also a non-significant effect [*F*_(1,1798)_ = 1.37, *p* = 0.24].

#### Other analyses

Repeating the analyses for both HDDs and DDs using a 3-level genotype variable yielded a non-significant trend for a moderating effect of the 3-level genotype on topiramate’s effects on HDDs (*p* = 0.06) (see Supplementary Methods, Results, and Supplementary Figs. S4 and S5).

### Impact of missing data

A pattern mixture analysis using a binary indicator of complete data as a summary of missing data showed results very similar to those reported above: for HDDs, the interaction between medication and the missing data indicator was non-significant [*F*_(1,1797)_ = 1.50, *p* = 0.22], with the placebo group having 1.66 (95% CI = (1.28, 2.15), *t*_1797_ = 3.81, *p* = 0.001) times more HDDs per week than the topiramate group. Interactions between the pattern variable and medication [*F*_(1,1797)_ = 1.44, *p* = 0.23], genotype [*F*_(1,1797)_ = 0.001, *p* = 0.96], and the medication by genotype interaction [*F*_(1,1797)_ = 0.61, *p* = 0.44] were all nonsignificant, and the medication by genotype interaction remained non-significant after adjustment for missing data [*F*_(1,1797)_ = 0.02, *p* = 0.89].

### Adverse effects

Two patients in the study experienced a serious adverse event: one patient in the topiramate group required hospitalization due to an exacerbation of asthma and one patient in the placebo group was hospitalized following a severe alcohol-related relapse. Both patients recovered fully.

Similar numbers of patients in the two medication groups reported experiencing at least one AE: 80 (94.1%) in the topiramate group and 76 (89.4%) in the placebo group [*χ*^2^_(1)_ = 1.25, *p* = 0.26]. Among patients reporting at least 1 AE, topiramate patients reported significantly more AEs (mean = 3.5, SD = 1.7) than placebo patients (mean = 2.6, SD = 1.3) [*χ*^2^_(1)_ = 8.90, *p* = 0.003]. Of the AEs reported, 85.7% were rated as mild, 13.7% as moderate, and 0.7% as severe. Topiramate patients reported more moderate or severe AEs (mean = 1.0, SD = 1.6) than placebo patients (mean = 0.4, SD = 0.8) [*χ*^2^_(1)_ = 9.94, *p* = 0.002]. The AEs that occurred in at least 10% of the patients and the number of patients from each group that experienced the event are summarized in Supplementary Table 1. A significantly greater number of topiramate patients than placebo patients reported paresthesias, dysgeusia and speech and language difficulties.

## Discussion

In this study, we replicated the finding that topiramate at a maximal daily dosage of 200 mg is efficacious in reducing heavy drinking in patients seeking either to reduce their drinking or become abstinent from alcohol. Findings for both GGT concentration, a biomarker of heavy drinking, and the Short Index of Problems score, which measures alcohol-related problems, corroborated the self-reported drinking data. The effects of topiramate in the present study were similar to those reported by us in our previous placebo-controlled trial of topiramate [10] and of a magnitude similar to the effect size in a meta-analysis of topiramate treatment trials [4]. In contrast to a key finding in our prior study, however, here the moderating effect of rs2832407 on topiramate’s efficacy, although in the hypothesized direction, was not statistically significant. Despite the absence of a pharmacogenetic effect, there was a reduction in the topiramate group of 1–2 HDDs/week. This effect was about 50% greater than in the placebo group and is clinically important, as the frequency of heavy drinking is correlated with a variety of alcohol-related negative consequences [20–22]. Thus, the findings reported here support the use of topiramate as a first-line treatment for AUD.

Based on these findings, it is possible that the pharmacogenetic effect seen in the prior study [10] was due to chance. The selection of a single SNP as a potential moderator was based on its association with AUD in a large case-control study [9]. However, the SNP has not been identified in any genome-wide association studies of AUD [23–25], suggesting that the initial finding may have been spurious. Of note, however, is that the moderating effect of rs2832407 in the present study though not statistically significant, was in the hypothesized direction, raising the possibility that the effect size in the initial report [10] was overestimated and the current study was underpowered to detect it. In this event, a larger study could show a significant interaction effect. A potential effect supporting this interpretation was seen when we repeated the analyses using a 3-level genotype variable, which yielded a non-significant, trend-level moderating effect on topiramate’s reduction of HDDs. However, randomization in the study was stratified by A/A + A/C versus C/C genotypes, so the comparisons of topiramate within the three-level genotype variable are not protected by randomization.

Although treatment response is partially genetically determined [26], it is a complex trait and thus is influenced by multiple genetic variants of small effect [27]. Despite evidence that statistical power is enhanced in the study of treatment‐relevant variants relative to disease-related ones [28], it may be unduly optimistic to expect that a single, non-coding SNP could serve clinically as a moderator of treatment response, particularly if the effect is a pharmacodynamic one. Pharmacokinetic effects of single gene variants, particularly gene deletions and duplications, on both treatment outcome and adverse effects, have been observed, for example in treating major depression with an antidepressant [29]. However, these variants are structural, often involving the deletion or duplication of a gene, and are more readily linked to metabolic effects on drug exposure than non-coding SNPs such as rs2832407.

Future efforts to identify genetic moderators of pharmacotherapy response in AUD should focus on genome-wide analyses. Because such efforts require large samples of patients treated with a specific medication, the time and expense for which can be prohibitive, an electronic health record approach may be required. Such an approach was used to examine the effects of gabapentin on alcohol consumption using repeated measurements of the Alcohol Use Disorders Identification Test-Consumption score [30]. This approach could be paired with genome-wide genotype data in a sample such as that in the Million Veteran Program [31] to identify variants, which could be tested individually or as polygenic risk scores in prospective RCTs.

## Funding and disclosure

Supported by grant AA023192 from the National Institute on Alcohol Abuse and Alcoholism and the Mental Illness Research, Education, and Clinical Center of the Veterans Integrated Service Network 4, U.S. Department of Veterans Affairs. PEM, TP, RCC, KMK, EEH, and KGL have no disclosures to make. HRK is a member of a Dicerna scientific advisory board and a member of the American Society of Clinical Psychopharmacology’s Alcohol Clinical Trials Initiative, which during the past 3 years was supported by AbbVie, Alkermes, Dicerna, Ethypharm, Indivior, Lilly, Lundbeck, Otsuka, Pfizer, Arbor Pharmaceuticals, and Amygdala Neurosciences, Inc. HRK is also named as an inventor on PCT patent application #15/878,640 entitled: “Genotype-guided dosing of opioid agonists,” filed January 24, 2018.

## Supplementary information

Supplemental Material
