# Correction to: Prospective randomized pharmacogenetic study of topiramate for treating alcohol use disorder

**DOI:** 10.1038/s41386-021-01013-6

**Published:** 2021-04-21

**Authors:** Henry R. Kranzler, Paige E. Morris, Timothy Pond, Richard C. Crist, Kyle M. Kampman, Emily E. Hartwell, Kevin G. Lynch

**Affiliations:** 1 Department of Psychiatry, University of Pennsylvania Perelman School of Medicine, Philadelphia, PA USA; 2 Mental Illness Research, Education and Clinical Center, Veterans Integrated Service Network 4, Crescenz VAMC, Philadelphia, PA USA

**Keywords:** Medical research, Diseases

Correction to: *Neuropsychopharmacology* 10.1038/s41386-020-00945-9, published online 10 February 2021

The article “Prospective randomized pharmacogenetic study of topiramate for treating alcohol use disorder”, written by Henry R. Kranzler, Paige E. Morris, Timothy Pond, Richard C. Crist, Kyle M. Kampman, Emily E. Hartwell and Kevin G. Lynch, was originally published electronically on the publisher’s internet portal on 10 February 2021 without open access. With the author(s)’ decision to opt for Open Choice the copyright of the article changed on 1^st^ April 2021 to © The Author(s) 2021 and the article is forthwith distributed under a Creative Commons Attribution 4.0 International License, which permits use, sharing, adaptation, distribution and reproduction in any medium or format, as long as you give appropriate credit to the original author(s) and the source, provide a link to the Creative Commons licence, and indicate if changes were made. The images or other third party material in this article are included in the article’s Creative Commons licence, unless indicated otherwise in a credit line to the material. If material is not included in the article’s Creative Commons licence and your intended use is not permitted by statutory regulation or exceeds the permitted use, you will need to obtain permission directly from the copyright holder. To view a copy of this licence, visit http://creativecommons.org/licenses/by/4.0/

