# Supplementary material for: Prospective randomized pharmacogenetic study of topiramate for treating alcohol use disorder
Source: Neuropsychopharmacology. 2021 Feb 10;46(8):1407–13. doi: 10.1038/s41386-020-00945-9 (PMC8209023; doi:10.1038/s41386-020-00945-9)

**Supplemental Methods:** We repeated the analyses of heavy drinking days and drinking days with genotype as a 3-level variable, as in our original publication (Kranzler et al. 2014), following the same approach as in the main analyses. Of note, we view these analyses as exploratory, as the study was not designed to examine this question, because the randomization was stratified by A/A+A/C versus C/C genotypes, so the comparisons of topiramate within the 3-level genotype variable are not protected by randomization. The 2-level genotype was chosen *a priori* based on the small number of rs2832407*A/A participants in the initial study, which was consistent with the allele frequencies in European Americans. In fact, only 22 of the 170 participants in the present study (i.e., 13%) were in this genotype group, with an unbalanced allocation to medication condition: n=14 in the topiramate group and n=8 in the placebo group. Thus, the results involving the A/A groups should be interpreted with caution.

**Supplemental Results:**

Moderating Effects of the 3-Level Genotype on the Response to Topiramate

*Heavy Drinking Days* (Supplemental Figure 4): At endpoint, within the C/C genotype group, participants receiving placebo reported 1.73 [95% CI=(1.13, 2.67)] times more heavy drinking days per week than those treated with topiramate, compared to 1.99 [95% CI=(1.39, 2.86)] times more heavy drinking days per week for placebo than topiramate within the A/C genotype group, and 1.38 [95% CI=(0.65, 2.92)] times fewer heavy drinking days per week for placebo than topiramate within the A/A genotype group, yielding a non-significant genotype by treatment interaction effect [F_(2,1797)_=2.84, p=0.06].

*Drinking Days* (Supplemental Figure 5): Among CC genotype patients, those treated with placebo reported 1.29 [95% CI=(1.03, 1.62)] times more drinking days per week than those receiving topiramate, compared to 1.16 [95% CI=(0.96, 1.40)] times more drinking days per week in the A/C genotype group, and 1.12 [95% CI=(0.75, 1.66)] times fewer drinking days per week in the A/A genotype group, yielding a non-significant genotype by treatment interaction effect. [F_(2,1799)_=1.28, p=0.28].

|  | | Numbers of Patients Reporting an Adverse Event | | | Statistics | | | | |  |  |
| --- | --- | --- | --- | --- | --- | --- | --- | --- | --- | --- | --- |
| Adverse Event | Overall Sample | | Placebo Group | Topiramate Group | | Chi- square | P-Value | Odds Ratio | OR Lower Limit | | OR Upper Limit |
| **Paresthesias** | **55** | | **9** | **46** | | **39.34** | **0.00** | **9.96** | **4.42** | | **22.43** |
| Headaches | 36 | | 15 | 21 | | 1.27 | 0.26 | 1.53 | 0.73 | | 3.22 |
| **Dysgeusia** | **35** | | **5** | **30** | | **24.47** | **0.00** | **8.73** | **3.19** | | **23.89** |
| Pain and discomfort | 33 | | 17 | 16 | | 0.04 | 0.85 | 0.93 | 0.43 | | 1.98 |
| Fatigue | 31 | | 10 | 21 | | 4.86 | 0.03 | 2.46 | 1.08 | | 5.61 |
| Upper resp infections | 31 | | 10 | 21 | | 4.86 | 0.03 | 2.46 | 1.08 | | 5.61 |
| Eye Disorders | 24 | | 7 | 17 | | 4.98 | 0.03 | 2.79 | 1.09 | | 7.12 |
| **Speech/Language abnormalities** | **21** | | **3** | **18** | | **13.40** | **0.00** | **7.34** | **2.07** | | **26.00** |
| Insomnia | 20 | | 8 | 12 | | 0.91 | 0.34 | 1.58 | 0.61 | | 4.09 |
| Somnolence | 18 | | 6 | 12 | | 2.28 | 0.13 | 2.16 | 0.77 | | 6.06 |
| Diarrhea | 17 | | 9 | 8 | | 0.07 | 0.80 | 0.88 | 0.32 | | 2.39 |
| Injuries NEC | 17 | | 8 | 9 | | 0.07 | 0.80 | 1.14 | 0.42 | | 3.11 |

**Supplemental Table 1: Counts and Comparisons for Adverse Events Endorsed by 10% or More of Patients**

Adverse events in bold are significant at a Bonferroni-corrected p-value of 0.0045, controlling for 11 comparisons

**Supplemental Figure 1: CONSORT Diagram**


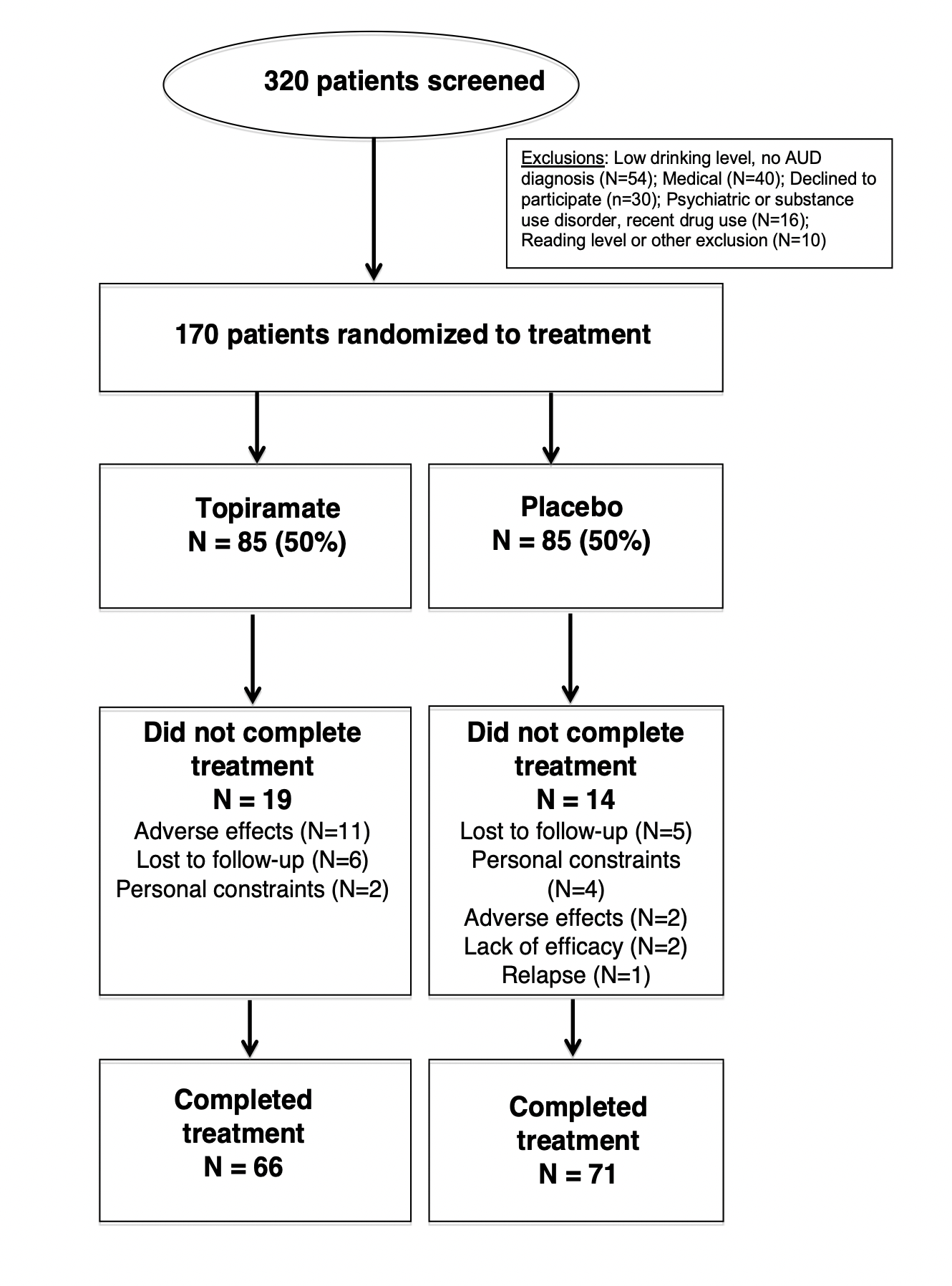


## Supplemental Figure 2: Mean (SEM) Abstinent Days per Week by Medication Group. There was a significant (linear) effect of week (F_(1,1798)_=11.50, p=0.0007) and a significant effect of medication group (F_(1,1798)_=4.52, p=0.03), with placebo group reporting 1.56 [95% CI=(1.18, 2.08)] times more drinking days per week than topiramate group.


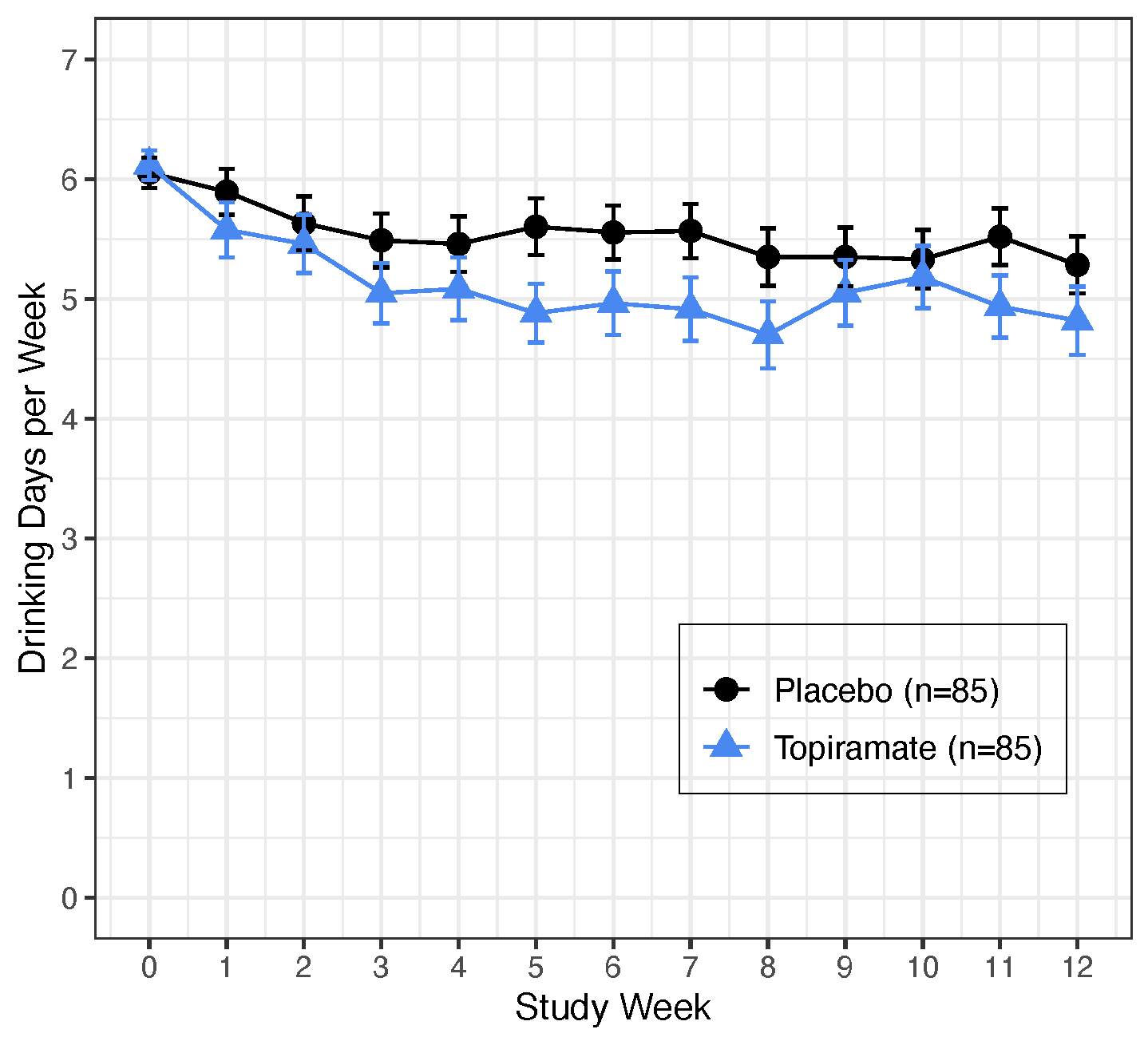


**Supplemental Figure 3: Mean (SEM) Abstinent Days per Week by Medication and Genotype Groups.** Panel A: At endpoint, in the CC genotype group, placebo patients reported 1.28 [95% CI=(1.02, 1.61)] times more drinking days per week than topiramate patients. Panel B: In the AC/AA genotype group, placebo patients reported 1.10 [95% CI=(0.93, 1.30)] times more drinking days per week than topiramate patients. The interaction effect was non-significant [F_(1,1798)_=1.16, p=0.28].


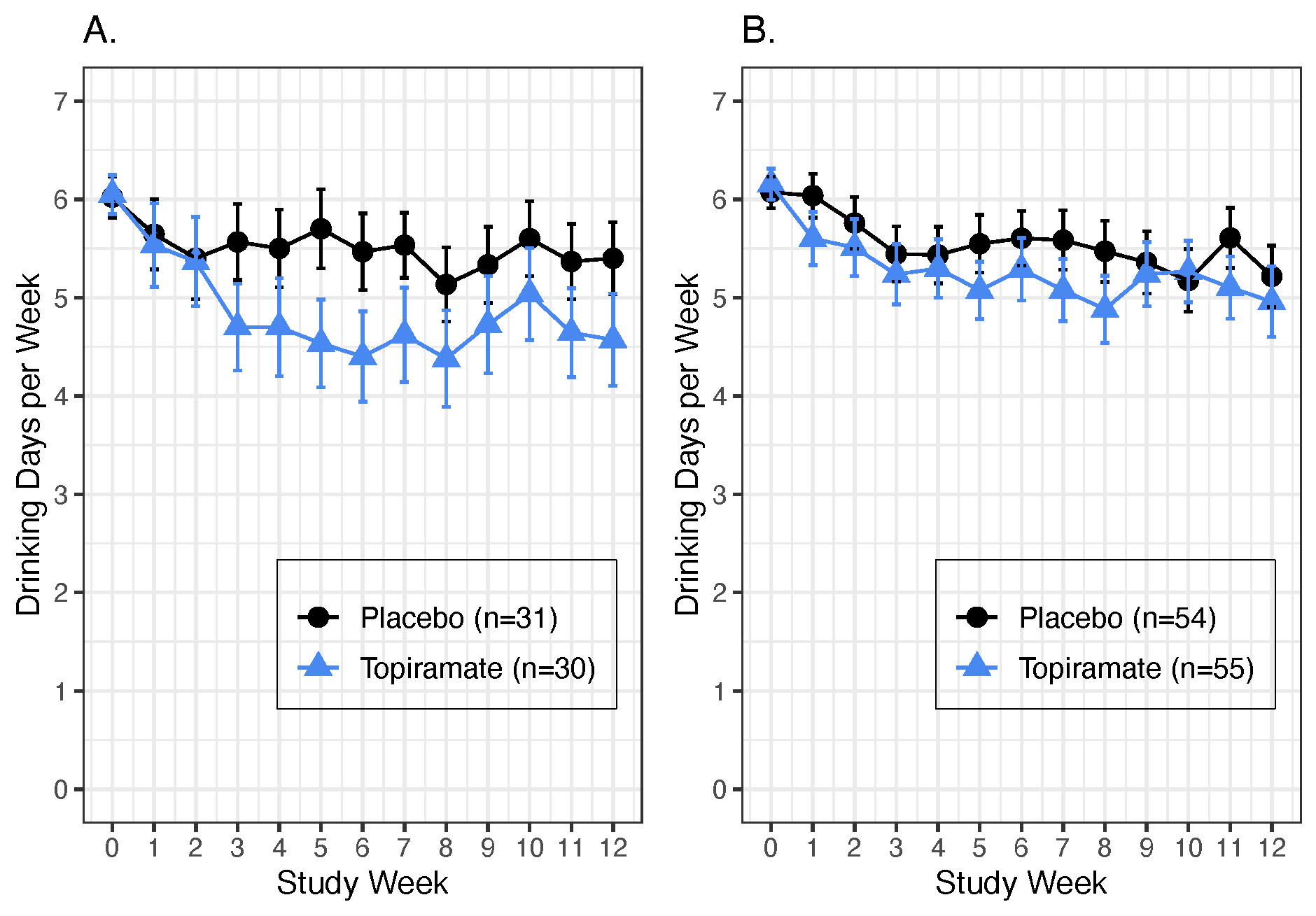


**Supplemental Figure 4: Mean (SEM) Heavy Drinking Days per Week by Medication and 3-Level Genotype Groups.** At endpoint, within the C/C genotype group (Panel A), participants receiving placebo reported 1.74 [95% CI=(1.13, 2.67)] times more heavy drinking days per week than those treated with topiramate. Within the A/C genotype group (Panel B), placebo-treated patients reported 1.98 [95% CI=(1.38, 2.84)] times more heavy drinking days per week than topiramate. Among participants in the A/A genotype group (Panel C), those receiving placebo reported 1.37 [95% CI=(0.65, 2.91)] times fewer heavy drinking days per week than the topiramate group. There was a non-significant genotype by treatment interaction effect [F_(2,1797)_=2.80, p=0.06].


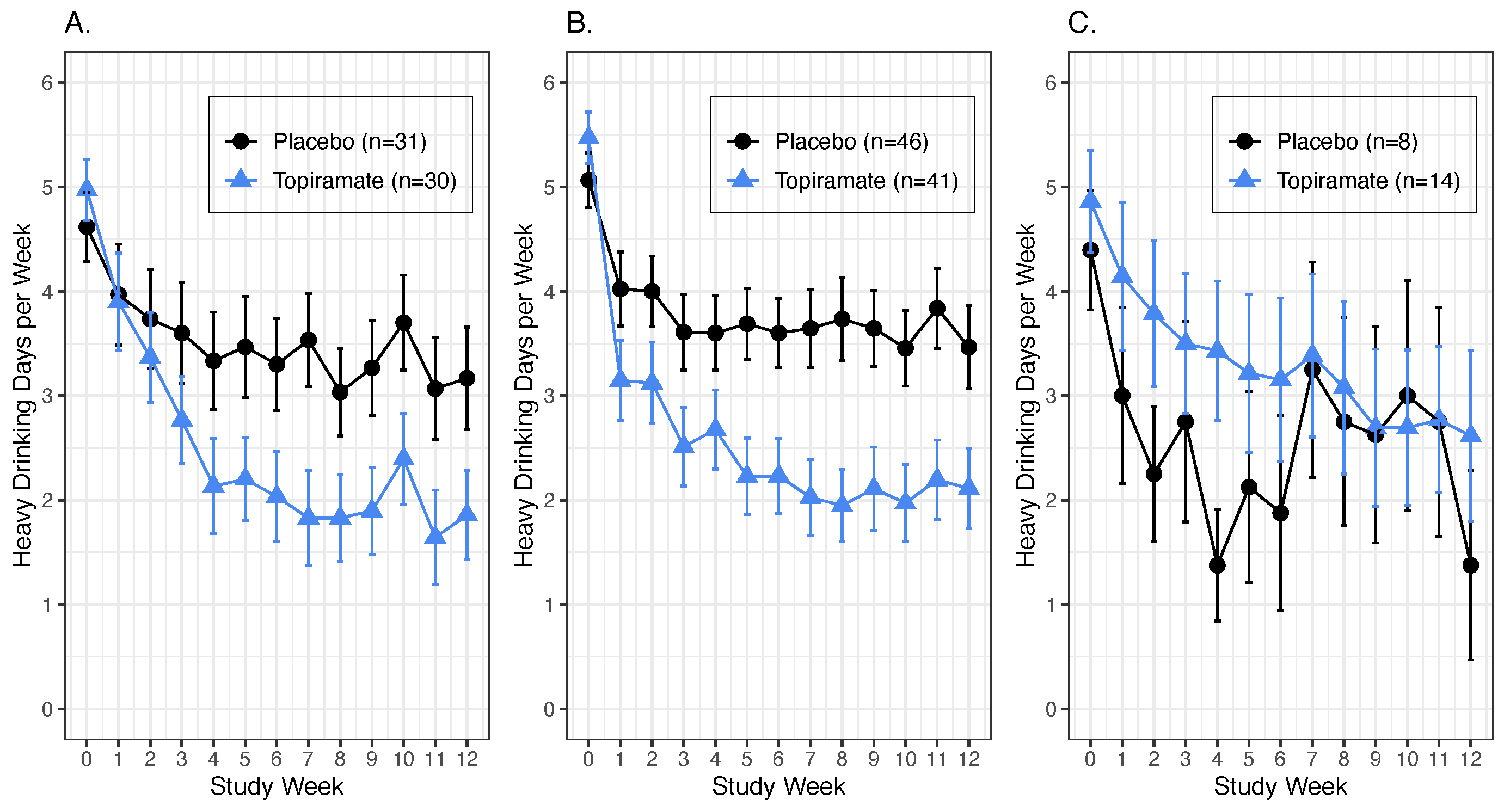


**Supplemental Figure 5: Mean (SEM) Drinking Days per Week by Medication and 3-Level Genotype Groups.** At endpoint, within the C/C genotype group (Panel A), participants receiving placebo reported 1.28 [95% CI=(1.02, 1.61)] times more drinking days per week than those treated with topiramate. Within the A/C genotype group (Panel B), placebo-treated patients reported 1.16 [95% CI=(0.95, 1.40)] times more drinking days per week than those receiving topiramate. Among participants in the A/A genotype group (Panel C), those receiving placebo reported 1.12 [95% CI=(0.76, 1.67)] times fewer drinking days per week than the topiramate group. The genotype by treatment interaction effect was not significant [F_(2,1799)_=1.22, p=0.30].


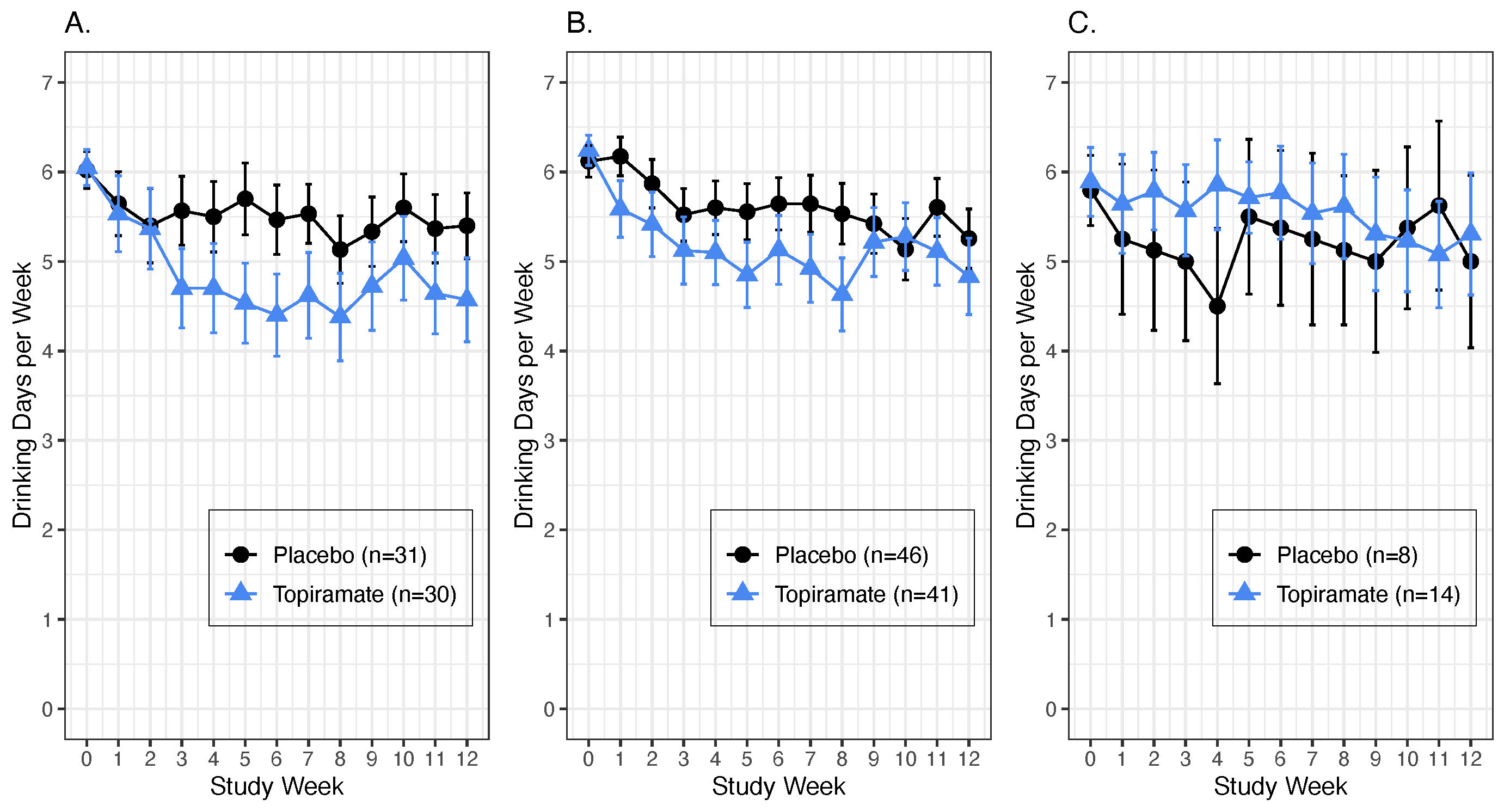

Supplement: Supplementary file 1 — Supplemental Material [file 41386_2020_945_MOESM1_ESM.docx]
